# Robot-Assisted Distal Pancreatectomy for Pancreatic Cancer with Type IIIa Portal Annular Pancreas Using a Novel Strategy of Ventral Pancreas Preservation: A Case Report

**DOI:** 10.70352/scrj.cr.25-0824

**Published:** 2026-04-16

**Authors:** Toru Noso, Masayoshi Hioki, Rentaro Doi, Kazuki Hara, Reo Shimomi, Seitaro Nishimura, Kento Mishima, Naoko Miura, Tetsuya Kagawa, Wataru Ishikawa, Yohei Kurose, Daisuke Nobuoka, Shinya Asami, Hiroshi Sadamori, Norihisa Takakura

**Affiliations:** Department of Surgery, Fukuyama City Hospital, Fukuyama, Hiroshima, Japan

**Keywords:** portal annular pancreas, robot-assisted distal pancreatectomy, postoperative pancreatic fistula, pancreatic transection

## Abstract

**INTRODUCTION:**

Portal annular pancreas (PAP) is a rare congenital anatomical variation, first reported in 1987, characterized by an uncinate process encircling the portal vein and fusing with the pancreatic body. While often asymptomatic, the presence of PAP poses a significant technical challenge during pancreatectomy because of its altered anatomy and the elevated risk of postoperative pancreatic fistula (POPF). Consensus is lacking on the optimal pancreatic transection method (1- or 2-cut margin approach) to minimize this complication. We report the first documented case of robot-assisted distal pancreatectomy (DP) for pancreatic cancer in a patient with PAP, highlighting a novel surgical strategy for pancreatic division.

**CASE PRESENTATION:**

A 75-year-old woman with pancreatic cancer was preoperatively diagnosed with PAP type IIIa (suprasplenic confluence). Following neoadjuvant chemotherapy, the patient underwent robot-assisted DP. A novel technique was executed using the robot’s stable, high-definition 3D vision and precise instrument control to dissect the ventral pancreas away from the dorsal pancreas, meticulously preserving the latter at the PAP fusion plane. The surgery achieved an R0 resection. Postoperatively, the patient’s course was favorable, with no clinically significant POPF. She was discharged on POD 11 and remained recurrence-free at 18 months.

**CONCLUSIONS:**

This case demonstrates the feasibility of a novel POPF-reducing transection strategy for challenging cases of PAP. Enabled by the robot’s stable 3D vision and precise control, this approach offers a new and viable surgical option for DP in patients with PAP type IIIa.

## Abbreviations

DP
: distal pancreatectomy
MPD
: main pancreatic duct
NAC
: neoadjuvant chemotherapy
PAP
: portal annular pancreas
POPF
: postoperative pancreatic fistula
PV
: portal vein
SA
: splenic artery
SV
: splenic vein

## INTRODUCTION

PAP is a rare pancreatic anatomical variation, in which the uncinate process of the pancreas encircles the PV and fuses with the dorsal surface of the pancreatic body. Although patients with PAP are often asymptomatic, the presence of this variation can cause difficulties during pancreatic resection due to the risk of POPF. A standard method of pancreatic division to reduce the risk of POPF has not yet been established.

Here, we present a case of robot-assisted DP in which the ventral pancreas was successfully dissected and preserved from the dorsal pancreas in a patient with PAP.

## CASE PRESENTATION

**Fig. 1:**
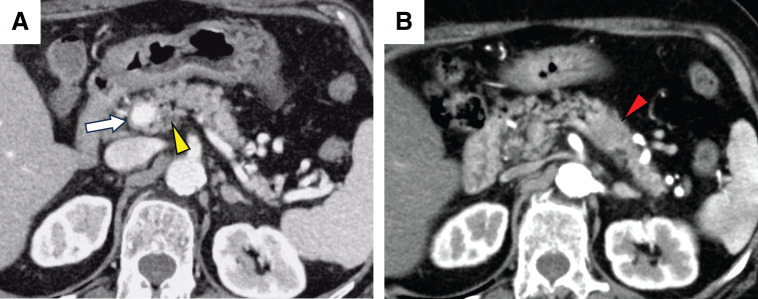
CT images. (**A**)–(**B**) are arranged in craniocaudal order. (**A**) The ventral pancreas (yellow arrowhead) encircles the PV (white arrow), consistent with a PAP. (**B**) A hypovascular tumor was observed at the pancreatic body–tail junction (red arrowhead). PAP, portal annular pancreas; PV, portal vein

**Fig. 2:**
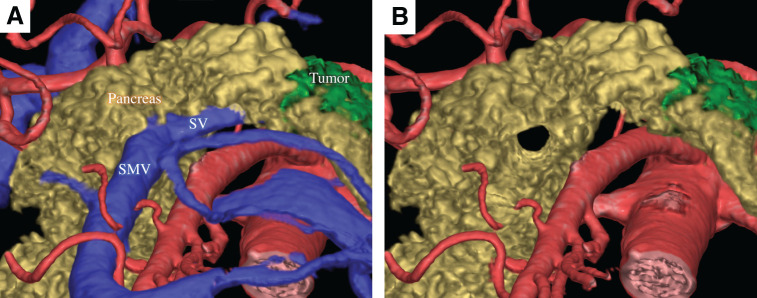
3D images created using SYNAPSE VINCENT (FUJIFILM, Tokyo, Japan). In image (**B**), where the portal system has been removed from image (**A**), the complete annular pancreas around the PV is clearly visible. PV, portal vein; SMV, superior mesenteric vein; SV, splenic vein

**Fig. 3:**
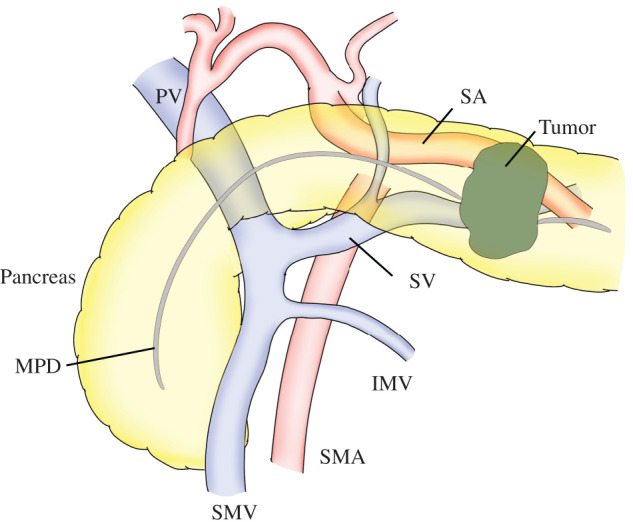
Preoperative anatomical relationship. IMV, inferior mesenteric vein; MPD, main pancreatic duct; PV, portal vein; SA, splenic artery; SMA, superior mesenteric artery; SMV, superior mesenteric vein; SV, splenic vein

A 75-year-old Japanese woman presenting with thirst and polyuria was referred to our hospital. Blood tests revealed elevated levels of carcinoembryonic antigen (6.5 ng/mL), carbohydrate antigen 19-9 (156 U/mL), and glycosylated hemoglobin (14.9%). She had a medical history of appendectomy for appendicitis at the age of 20 years, and was currently taking medication for hypertension. Endoscopic retrograde pancreatography revealed stenosis at the MPD in the pancreatic body with upstream dilation; however, pancreatic juice cytology was negative for malignancy. Endoscopic ultrasonography revealed a hypoechoic lesion (16 × 16 mm) in the pancreatic body, and fine-needle aspiration confirmed adenocarcinoma. Contrast-enhanced CT revealed a hypovascular tumor at the junction of the pancreatic body and tail and a PAP superior to the SV (**Fig. 1**). **Figure 2** shows the 3D images created using SYNAPSE VINCENT (FUJIFILM, Tokyo, Japan). No lymph node metastases or distant metastases were observed. The patient was diagnosed with pancreatic cancer with PAP. Diagnostic laparoscopy confirmed the absence of macroscopic liver metastases and peritoneal dissemination. The peritoneal lavage cytology results were negative. According to the Japanese classification of pancreatic carcinoma by the Japan Pancreas Society (8th edition),^1)^ the preoperative clinical staging was T3N0M0, Stage IIA. The pancreatic cancer was judged to be resectable, and surgery was planned after 2 cycles of gemcitabine and tegafur-gimeracil-oteracil potassium as NAC. Based on the positional relationship between the tumor and PV on preoperative imaging, we planned to transect the pancreas at the level of the PV, anticipating that this would provide a surgical margin of at least 3 cm from the tumor. However, the final transection line would be determined using intraoperative ultrasonography. Furthermore, because this patient had no history of pancreatitis and the MPD coursed ventral to the PV, we considered a method in which the fusion plane between the ventral and dorsal pancreas was dissected, allowing preservation of the ventral pancreas without transection. Finally, robot-assisted DP was planned. The positional relationships of the pancreas, vessels, and tumor are shown in **Fig. 3**.

**Fig. 4:**
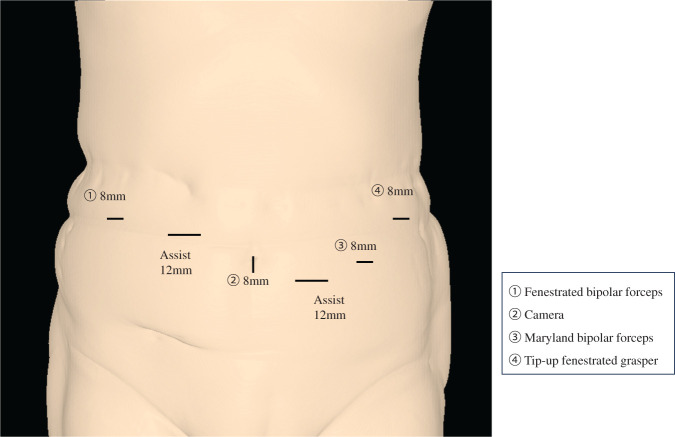
Trocar placement in robot-assisted DP. DP, distal pancreatectomy

**Fig. 5:**
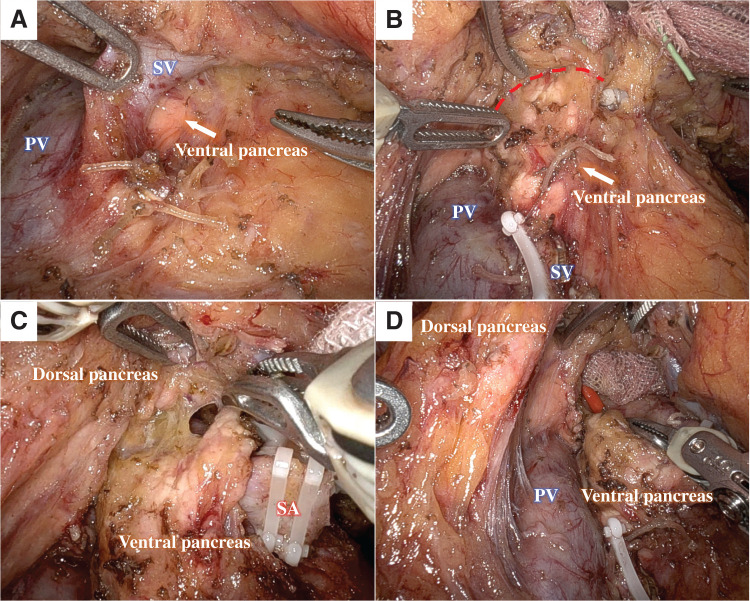
Intraoperative findings. (**A**) The SV-cephalad PAP. (**B**) and (**C**) A space was observed between the ventral and dorsal pancreatic parenchyma, and dissection was performed along the red dashed line. (**D**) Dissection was completed, leaving the most cephalad fusion plane intact. PAP, portal annular pancreas; PV, portal vein; SA, splenic artery; SV, splenic vein

Surgery was performed using the da Vinci Xi Surgical System (Intuitive Surgical, Sunnyvale, CA, USA). The operation began with a 6-port configuration (**Fig. 4**). The SA was exposed via the gastrohepatic ligament approach.^2)^ The SA was then ligated, clipped, and divided. The PV was exposed dorsally to the common hepatic artery. After opening the omental bursa, the pancreatic body, tail, and spleen were mobilized. The SV was identified by tunneling anterior to the superior mesenteric vein. The PAP was confirmed to lie cephalad to the SV (**Fig. 5A**), consistent with preoperative findings. The SV was subsequently ligated, clipped, and divided.

Upon close inspection of the PAP confluence, a narrow gap was visible between the dorsal and ventral pancreas parenchyma (**Fig. 5B**). Although the MPD was preoperatively known to run anterior to the PV, dissection was performed carefully to avoid injury to the duct or pancreatic parenchyma. The PAP was dissected primarily from the caudal to the cephalad direction (**Fig. 5C**). However, in the most cephalad region, the boundary between the ventral and dorsal pancreatic parenchyma was indistinct. As this had no impact on the planned pancreatic transection, the fusion plane was left intact (**Fig. 5D**). After confirming a sufficient margin from the tumor using intraoperative ultrasonography, the pancreas was compressed for 15 min at the level of the left edge of the PV. The pancreatic transection was then performed using the Endo GIA reinforced purple reload with Tri-Staple 60 mm (COVIDIEN, North Haven, CT, USA). Finally, the ventral pancreas was spared and preserved. A negative-pressure closed-suction drain was placed near the pancreatic stump, and surgery was completed.

The surgery time was 517 min, and the blood loss was minimal. Postoperatively, biochemical leakage, as classified by the International Study Group of Pancreatic Fistula,^3)^ occurred; however, the drain was removed on POD 4, and the patient was discharged on POD 11.

The pathological diagnosis was T1cN0M0, Stage IA, based on the Union for International Cancer Control 8th edition and T3N0M0, Stage IIA, based on the Japanese classification of pancreatic carcinoma by the Japan Pancreas Society (8th edition).^1)^ The therapeutic response to NAC was Grade 1b.^1)^ The tumor was resected with an adequate margin (40 mm), and an R0 resection was achieved. Adjuvant chemotherapy with tegafur-gimeracil-oteracil potassium was initiated on POD 38, and the patient is currently alive with no recurrence 18 months after surgery.

## DISCUSSION

PAP is an anatomical variation of the pancreas first reported by Sugiura et al.^4)^ in 1987. Embryologically, the human pancreas originates from 2 separate endodermal buds—ventral and dorsal—which rotate and fuse during the seventh week of gestation to form a single gland. The embryological development of the pancreas is complex, and various malformations are known to occur, including PAP, pancreas divisum, and an anomalous pancreaticobiliary junction. PAP is a malformation in which the ventral pancreatic bud wraps around the left side of the PV and fuses with the body of the pancreas during its rotation behind the foregut.^5,6)^ The reported prevalence of PAP ranges from 0.5% to 2.5%,^7,8)^ and case reports of pancreatectomy in patients with PAP are limited. To date, only 13 cases of DP have been reported in patients with PAP (**Table 1**).^9–19)^

**Table 1:** Reported 13 cases of DP in patients with PAPs

Author	Year	Age	Sex	Pancreatic disease	Classification of PAP	Method of surgery	No. of cut margins	POPF	Postoperative hospital stay (days)
Hashimoto^9)^	2009	39	F	MCN	I	Open	2	Yes (BL)	9
Jang^10)^	2012	74	F	IPMN	IIIa	Lap	2	Yes (BL)	16
Yamaguchi^11)^	2013	80	F	Pancreatic sarcoidosis	I	Open	2	No	–
Harnoss^12)^	2014	48	F	Suprarenal cancer	IIIa	Open	2	Yes (grade B)	13
Ohtsuka^13)^	2017	63	M	PNET	IIIa	Open	2	No	–
Ohtsuka^13)^	2017	61	F	PDAC	IIIa	Open	2	Yes (grade B)	–
Yuan^14)^	2017	74	M	PDAC	IIIb	Open	2	No	11
Kuriyama^15)^	2020	47	F	SCN	IIIa	Lap	2	Yes (grade B)	9
Abe^16)^	2021	70	M	Pancreatic cancer	II	Open	2	No	23
Polyakov^17)^	2023	33	F	Solid pseudopapillary tumor	IIIa	Lap	2	Yes (grade B)	6
Habu^18)^	2025	79	M	HCC LNM	IIIc	Lap	2	Yes (grade B)	33
Hakoda^19)^	2025	72	M	PDAC	IIIa	Open	2	No	11
Our case	2025	75	F	PDAC	IIIa	Robot	1	Yes (BL)	11

BL, biochemical leakage; DP, distal pancreatectomy; F, female; HCC, hepatocellular carcinoma; IPMN, intraductal papillary mucinous neoplasm; LNM, lymph node metastasis; Lap, laparoscopy; M, Male; MCN, mucinous cystic neoplasm; PAP, portal annular pancreas; PDAC, pancreatic ductal adenocarcinoma; PNET, pancreatic neuroendocrine tumor; POPF, postoperative pancreatic fistula; SCN, serous cystic neoplasm

In 2009, Karasaki et al.^20)^ classified PAP into 3 types based on its relationship with the SV and its confluence: supra-SV, infra-SV, and mixed types. Subsequently, Joseph et al.^5)^ introduced a classification based on the course of the MPD: type I, where the MPD courses dorsal to the PV; type II, which coexists with the pancreas divisum; and type III, where the MPD courses ventral to the PV. Furthermore, type III was subclassified according to its relationship with the SV: type IIIa, when the confluence was supra-SV; type IIIb, infra-SV; and type IIIc, mixed type. Pandrowala et al.^21)^ and Harnoss et al.^12)^ reported that type IIIa is the most frequent type, and the present case was classified as type IIIa according to Joseph’s classification.

PAP is usually asymptomatic and often does not pose a clinical problem; however, in the context of pancreatectomy, several considerations arise. Because PAP lies close to the PV, particular attention must be paid to avoiding intraoperative bleeding,^16)^ and the fused ventral pancreatic parenchyma can influence the transection line, potentially making it difficult to secure an adequate margin from the tumor. Among these considerations, the most important concern is the increased incidence of POPF. Ohtsuka et al.^13)^ reported that patients with PAP have a threefold higher incidence of pancreatic fistulas than those with a normal pancreas. Preoperative diagnosis of PAP and subsequent surgical simulation, which confirm the spatial relationships of the MPD, PV, and pancreatic parenchyma, are considered beneficial for reducing the incidence of POPF. Nonetheless, Harnoss et al.^12)^ reported that in 52.9% of surgical cases of PAP, the condition was not diagnosed preoperatively but was instead found during the operation. This underscores the challenges in the preoperative diagnosis and the prevailing lack of awareness regarding this condition.

The optimal pancreatic transection technique for pancreatectomy in patients with PAP remains unclear. Ohtsuka et al.^13)^ discussed 2 approaches: the 1-cut margin method, which involves pancreatic transection distal to the PAP, and the 2-cut margin method, which entails pancreatic transection at the anterior aspect of the PV and a separate division of the fused pancreatic parenchyma.

All 12 previous cases were treated using the 2-cut margin method. In reports that included a description of the transection procedure, the method involved securing the dorsal pancreas on the anterior surface of the PV and transecting it with a stapler, followed by dissecting around the ventral pancreas and completing the transection. No descriptions were found indicating that the fused plane of the PAP was dissected. Creating 2 transection surfaces is considered a risk factor for the development of POPF, and, in fact, Grade B POPF occurred in 5 cases (38%) (**Table 1**). Conversely, performing a single transection distal to the PAP tends to result in a larger cross-sectional area of the pancreatic stump, which similarly increases the risk of POPF. Indeed, Mendoza et al.^22)^ stated that a pancreatic transection line thickness of 12 mm or greater is a risk factor for POPF. Furthermore, in the case of DP, the 1-cut margin approach may raise oncological concerns because the transection line is positioned closer to the lesion, which is located more distally. Therefore, it is difficult to definitively recommend 1 method over another.

**Fig. 6:**
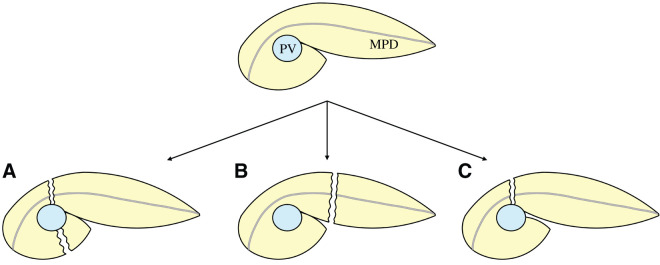
Three pancreatic transection options for type IIIa PAP. (**A**) 2-cut margin method. (**B**) 1-cut margin method. (**C**) The present method, in which the plane between the ventral and dorsal pancreas is dissected, allowing preservation of the ventral pancreas. MPD, main pancreatic duct; PAP, portal annular pancreas; PV, portal vein

Although we recognized that previous DP for PAP had been performed using the 2-cut margin method, we focused on the fact that the PAP consists of 2 distinct embryologic tissues: the ventral and dorsal pancreatic buds. Based on this concept, we preoperatively planned a new approach in which the ventral and dorsal portions of the PAP were dissected and only the dorsal portion was preserved, and we executed the surgery accordingly. This method offers advantages not seen in the conventional 2-cut margin or 1-cut margin method, namely that only a single pancreatic transection is required and the transection can be performed at a site with a smaller pancreatic cross-section (**Fig. 6**).

Furthermore, this case represents the first robot-assisted DP performed for a patient with PAP (**Table 1**). We consider that the features unique to robot-assisted surgery—specifically the stable instrument control and the high-quality visualization—enabled this surgical approach. In particular, the high-definition 3D view with a fixed operative field was essential for identifying the boundary between the ventral and dorsal pancreas, and the tremor filtration and motion scaling capability were indispensable for dissecting along this boundary without deviation. Robot-assisted DP has already been shown to have lower conversion rates and lower rates of failure in spleen preservation compared with laparoscopic DP,^23,24)^ suggesting an advantage in procedures requiring meticulous manipulation. Therefore, a technique that was previously difficult to perform via open or laparoscopic surgery has become feasible with the widespread adoption of robot-assisted surgery.

The main limitation of this study is that it remains unclear whether the technique used in this case—dissection and preservation of the ventral pancreas—can be generally adopted as a standard surgical procedure. This technique depends on the type of the PAP and degree of fusion between the ventral and dorsal pancreas, and in some cases the fusion plane may not be clearly identifiable, making dissection difficult. Excessive or forced dissection could damage the pancreatic parenchyma, and conversion to the conventional transection method may therefore be necessary. However, considering the increasing adoption of robot-assisted surgery in many institutions, we believe that this technique may eventually be recognized as one of the surgical options for patients with PAP, and that the accumulation of additional cases may allow the indications for this approach to be determined more accurately.

Herein, we present the first documented case of robot-assisted DP in a patient with PAP. As mentioned above, this case followed a favorable course without the development of clinically relevant POPF. As experience with this pancreatic transection method accumulates, it could become a viable option for DP in patients with PAP.

## CONCLUSIONS

In this case, we performed a novel robot-assisted DP technique in a high-POPF-risk situation associated with PAP involving dissection and preservation of the ventral pancreas from the dorsal pancreas. This may be a new surgical option for DP in patients with type IIIa PAP.
